# Extending the evaluation of Genia Event task toward knowledge base construction and comparison to Gene Regulation Ontology task

**DOI:** 10.1186/1471-2105-16-S10-S3

**Published:** 2015-07-13

**Authors:** Jin-Dong Kim, Jung-jae Kim, Xu Han, Dietrich Rebholz-Schuhmann

**Affiliations:** 1 Database Center for Life Science, Research Organization of Information and Systems, 178-4-4 Wakashiba, Kashiwa, Japan; 2 Nanyang Technological University, School of Computer Engineering, Block N4 #02a-32, Nanyang Avenue, Singapore 639798; 3 University of Zurich, Institute of Computational Linguistics, BinzmÃ¼hlestrasse 14, 8050 Zurich, Switzerland

**Keywords:** bionlp, shared task, evaluation, information extraction, text mining, knowledge base, semantic web, resource description framework

## Abstract

**Background:**

The third edition of the BioNLP Shared Task was held with the grand theme "knowledge base construction (KB)". The Genia Event (GE) task was re-designed and implemented in light of this theme. For its final report, the participating systems were evaluated from a perspective of annotation. To further explore the grand theme, we extended the evaluation from a perspective of KB construction. Also, the Gene Regulation Ontology (GRO) task was newly introduced in the third edition. The final evaluation of the participating systems resulted in relatively low performance. The reason was attributed to the large size and complex semantic representation of the ontology. To investigate potential benefits of resource exchange between the presumably similar tasks, we measured the overlap between the datasets of the two tasks, and tested whether the dataset for one task can be used to enhance performance on the other.

**Results:**

We report an extended evaluation on all the participating systems in the GE task, incoporating a KB perspective. For the evaluation, the final submission of each participant was converted to RDF statements, and evaluated using 8 queries that were formulated in SPARQL. The results suggest that the evaluation may be concluded differently between the two different perspectives, annotation vs. KB. We also provide a comparison of the GE and GRO tasks by converting their datasets into each other's format. More than 90% of the GE data could be converted into the GRO task format, while only half of the GRO data could be mapped to the GE task format. The imbalance in conversion indicates that the GRO is a comprehensive extension of the GE task ontology. We further used the converted GRO data as additional training data for the GE task, which helped improve GE task participant system performance. However, the converted GE data did not help GRO task participants, due to overfitting and the ontology gap.

## Background

The BioNLP Shared Task (BioNLP-ST) has been organized three times since 2009. The goal is to provide the community with shared resources for the development and evaluation of fine-grained information extraction (IE) systems, particularly for the domain of molecular biology and medicine. Each time, it was organized with a grand theme (a goal shared by all the tasks): *introduction of the event extraction task, generalization*, and *knowledge base (KB) construction*, for the 1st, 2nd, and 3rd editions, respectively [1-3].

Initially motivated by the *Genia annotation *[4], the tasks of BioNLP-ST are designed for *intrinsic evaluation*, with the hope of complementing more application-oriented tasks of *extrinsic evaluation*, e.g. the Protein-Protein Interaction (PPI) extraction task of BioCreative [5]. While an extrinsic evaluation measures the performance of a system in the context of a specific application, i.e. its utility in the entire application, an intrinsic evaluation focuses on measuring the performance of a system in isolation, independently from a specific application [6]. For example, while the BioCreative PPI task is explained in the context of database curation specifically, the potential application of BioNLP-ST tasks is often broadly explained.

Until its second edition, the participants in BioNLP-ST tasks were evaluated from a perspective of *annotation: *The annotation instances in the submitted and gold annotations are individually compared for evaluation. For its third edition in 2013, BioNLP-ST attempted to broaden the scope of its potential applications to include *KB construction*, which is expected to be closer to the interest of general domain scientists, e.g. biologists, and set it as its grand theme.

Among the tasks organized in BioNLP-ST, the Genia Event (GE) task is the sole task that has continued from the beginning [7-9]. For its third edition, the grand theme - *KB construction *- was considered in the design and implementation of the task: the coreference task [10] was integrated, for improved sensitivity of knowledge harvesting, and recently published full papers were added to the benchmark dataset [9]. However, the evaluation of the participating systems was still carried out in the same way as previously, in the context of corpus annotation, without implementing the grand theme into it much.

**Figure 1:**
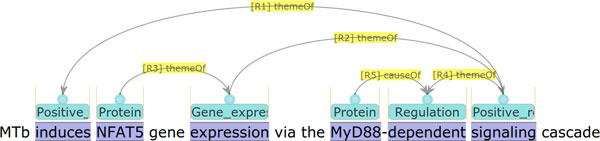
**Annotation example 1 in visualization**. MTb induces NFAT5 gene expression via the MyD88-dependent signaling cascade

**Figure 2:**
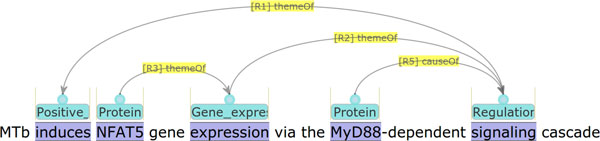
**Annotation example 2 in visualization**.

The work presented in this paper addresses extension of the GE task evaluation, considering KB construction as a potential application of the task. Specifically, what is intended with the new evaluation is to measure the effect of abstracting out schematic differences in annotation, which is not a concern of domain scientists, but rather of annotation practitioners. An example of schematic differences in annotation is illustrated in Figures 1 and 2. The example text reads that the signaling cascade that involves the protein *MyD88 *induces the expression of the protein *NFAT5*. However, the interpretation is represented differently in annotations in the two figures. In Figure 1, the two words *signaling *and *dependent *are annotated as triggering two successive regulation events, whereas in Figure 2, the word *dependent *is not annotated as triggering a regulation event, but as connecting the protein *MyD88 *to the signaling cascade as a causal factor. To annotation practitioners, and those who are interested in automating the annotation, this is an important issue, as it is related to the consistency of the annotations. This is the perspective from which the original evaluation is performed. We call it *annotation-oriented evaluation*. Domain scientists, however, would not be interested in such a difference, and would want to avoid being affected by it during their use of a KB. This is the perspective of the new evaluation, which we call *KB-oriented evaluation*.

The paper reports the results of the KB-oriented evaluation on all of the final submissions to the GE task in 2013. It complements the overview paper for the task [9], which provides a general introduction to the task and reports the results of the annotation-oriented evaluation.

This paper also provides a comparison of the GE task to the Gene Regulation Ontology (GRO) task [11], which is to automatically annotate biomedical documents with the Gene Regulation Ontology [12]. GRO is a conceptual model of gene regulation. It includes 507 concepts, which are cross-linked to such standard ontologies as Gene Ontology and Sequence Ontology and are integrated into a deep hierarchical structure via is-a and part-of relations. It is much larger than the Genia ontology, and its concepts are generally more specific than the Genia ontology concepts used in the previous GE tasks.

The complex structure of the GRO enables us to evaluate participant systems at different abstraction/generalization levels. However, its large size and complex semantic representation make the event extraction based on the ontology highly challenging. One of the issues of the GRO task is that its dataset is small compared to the size of the ontology. In this paper, we test whether the conversion of the GE task dataset, whose ontology has a large overlap with the GRO, into the GRO task may help address this issue.

## Methods

### Representation

Since its first edition in 2009, the annotation of the BioNLP-ST has been provided in the so-called a* format. In addition to the a* format, in the third edition of the GE task, the datasets are also provided in a new format, which is motivated by the following two issues.

**1. For ease of implementation: **The a* format is actually a quite complex format for which to implement reader and writer modules: it has to handle three delimiter characters, *tab, space*, and *colon*, in a structural way, and has to handle coordination of arguments based on number suffixes. The complexity is an extra overhead that has nothing to do with the task itself. The burden of the implementation prevents the participants from concentrating on the task. Using a more standard format would let them spend more time on the task itself.

**2. For flexible representation and retrieval of information: **As the a* format requires any piece of information related to an event to be represented in an event-centric n-ary relationship, it is hard to represent partial information. For example, even if there is a system which is very good at extracting causal relationships, since the information cannot be represented without successfully extracting some other part, e.g. the theme, of the relevant event, there is no way to evaluate the potential of the system. Also, from a KB perspective, the n-ary relationship makes the representation unsuitable to inference, which is necessary to provide flexible access to the contents of a KB. For example, from the annotation in Figures 1 and 2, users may want to retrieve the information that the protein *MyD88 *is a causal factor of *NFAT5 gene expression*, regardless of how it is represented in annotation, which, however, requires inference over the explicit annotation. To fulfill a KB-oriented use case, a more inference-friendly representation would be beneficial.

To address these issues, we present a new format. It is a JSON application. Note that JSON is currently one of the most widely used standard data formats and most major programming languages already have public reader and writer modules for it. Being provided with the benchmark dataset in JSON, the developers do not need to implement reader and writer modules themselves. The new format is also designed to be *relation-centric*, and all the information is represented by binary relations, which are more elementary than events. The new format is thus flexible enough to represent various aspects of information, and it is inference-friendly.

**Figure 3:**
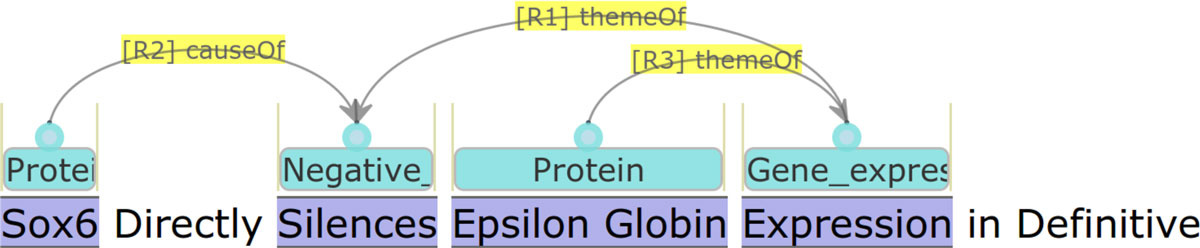
**Annotation example 3 in visualization**.

**Figure 4:**
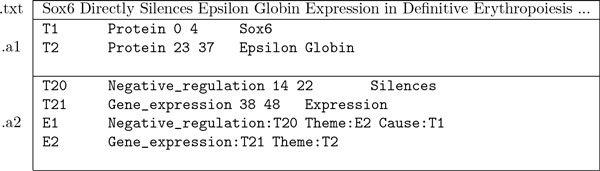
Annotation example 3 in BioNLP a* format.

For example, the annotation illustrated in Figure 3 is represented in the a* format as shown in Figure 4. The format is basically a variation of the CSV (comma-separated values) format with the *tab *character as the the primary delimiter. In the format, the annotation statements, which are n-tuples, are in the second column, while the ID of each statement is in the first column. The statements which have their ID beginning with the prefix *T *are *entity annotations*, and they are in the form of triples, (entity-type, beginning-caret-offset, ending-caret-offset), which state that the text span between the beginning- and ending-caret-offsets  denotes an entity of the entity-type. In the a* format, the elements of the triple are delimited by the *space *character. The statements which have their ID beginning with the prefix *E *are *event annotations*. They are in the form of typed n-tuples, (event-type:trigger-entity-id, arg1-type:arg1-entity-id, ...), representing the predicate-argument structure of an event. The order of the tuple, n, varies according to the number of arguments. Note that in the example, the event *E1 *has two arguments, *E2 (Theme) *and *T1 (Cause)*. As the a* format is event-centric, the event is represented as one statement representing its predicate-argument structure, regardless of the number of arguments involved in it.

**Figure 5:**
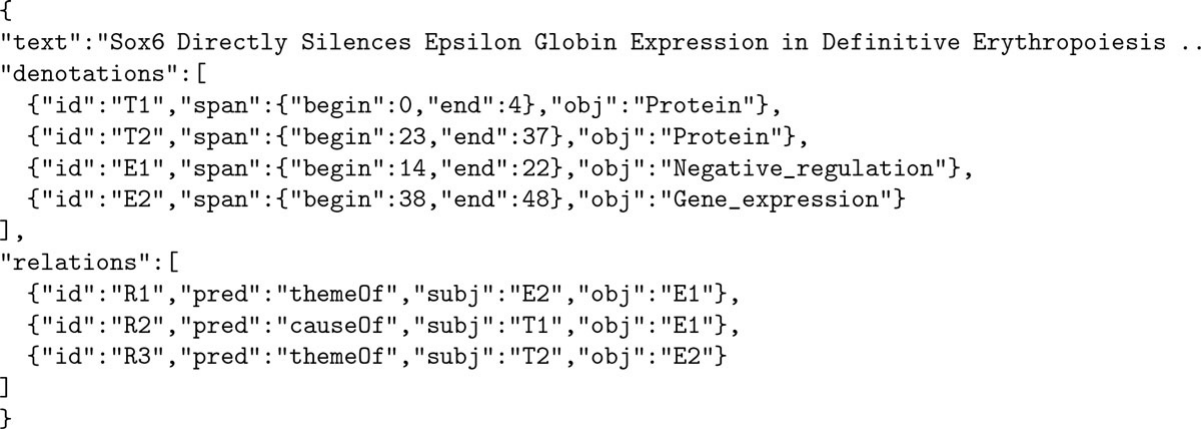
**Annotation example 3 in JSON**.

Figure 5 shows the same annotation in the JSON format. As can be seen, the JSON format is more self-descriptive than the a* format. A *denotation *type of annotation, which is stored in the array *denotations*, states that a span of text "denotes" an object. The *relation *type of annotation, which is stored in the array *relations*, states that two objects, of which one is *subject *and the other is *object*, are related to each other via a *predicate*. Note that the JSON format is relation-centric: a relation is represented by a single statement. In the example, the two (binary) relations, *R1 *and *R2*, together with the denotation, *E1*, correspond to the event *E1 *in the a* format. Such a conversion from n-ary relation to n binary relations is a standard process in description logic [13]. As a relation can be represented individually, a partial piece of information about an event, e.g. the causal relation, *R2*, can be represented independently from the other relation, *R1*, of the event. Through inference over relations, implicit information also can become accessible. For example, by defining the relation *themeOf *as a transitive one, it becomes straightforward to access the information that the protein *Sox6 *negatively regulates the protein *Epsilon Globin*. Note that the denotation annotations also can be seen as a special case of relation annotations, with text spans as their subjects. For more detailed information on the JSON format, readers are referred to the web document [14].

Considered at an abstract level, the pros and cons of the two formats become clearer. The a* format is closer to the relational database (RDB) model, i.e. tables, and the JSON format is closer to the resource description framework (RDF) model, i.e. graphs. The former is often more efficient for optimized applications, e.g. event-centric processing, while the latter is more flexible and inference-friendly. The GE task supports both models by providing the benchmark dataset in both formats, and also tool for converting between them.

### KB construction

To evaluate the annotation submitted by the participants from the perspective of KB construction, we built a KB from the annotations of each participant. As the framework of the KB, we have chosen to use the Resource Description Framework (RDF) [15], a widely accepted knowledge representation framework recommended by the World Wide Web Consortium (W3C) [16].

As the vocabulary for RDF statements, some existing open vocabularies were considered [17,18]. However, we found that they are focused on retaining provenance of information. As our purpose of constructing the KBs in this work is for evaluation of annotation, we have chosen to develop a minimal in-house vocabulary, which we call *text annotation ontology (TAO)*. Then, we implemented a converter from the JSON format to RDF. In fact, the JSON format is very close to RDF, as each annotation statement is already a triple. Converting it to RDF is explicating its semantics using an RDF vocabulary.

**Figure 6:**
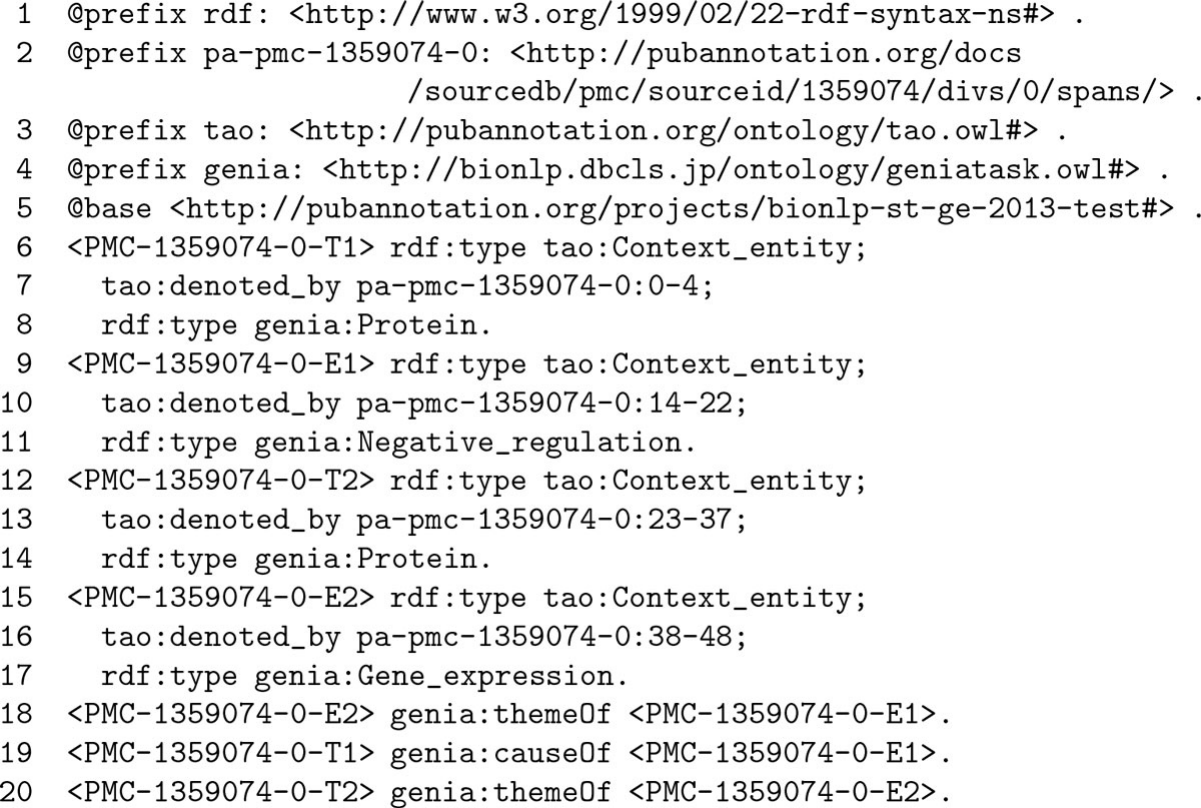
**Annotation example 3 in RDF**.

Figure 6 shows an annotation example in RDF using TAO. As can be seen in lines, 6, 9, 12, and 15, each denotation annotation introduces an entity which is of the type tao:Context_entity. As the name implies, an instance of the class tao:Context_entity  is an entity defined in a specific context, denoted by a span of text. The predicate tao:denoted_by  connects a context entity, as the subject, to a span of text, as the object. It is an inverse predicate of tao:denote. The URLs used for the span specification, e.g. http://pubannotation.org/docs/sourcedb/PMC/sourceid/1359074/divs/0/spans/0-4, are dereferenceable ones which are provided by the PubAnnotation service [19]. Each relation annotation is simply represented by a statement using either of the predicates in the GE task namespace, e.g., genia:themeOf , or genia:causeOf .

Using the vocabulary, the annotation submitted by each of the 10 participants has been converted into a RDF graph, which then has been loaded into a RDF store.

## Queries

To evaluate the 10 KBs constructed from the final submissions, 8 queries were prepared in SPARQL, as shown in Table 1. The queries are designed to demonstrate the effect of abstracting out schematic differences in annotation. We consulted with biologists and bioinformaticians to prepare queries useful to domain scientists, within the scope of the GE task. However, this is not meant to be an extrinsic evaluation, and the set of queries is not comprehensive. Rather, it is meant to be an intrinsic evaluation, to highlight focused features of a system. Each of the prepared queries thus has its own goal, which is described below.

**Table 1:** Queries used for the KB evaluation.

#	Meaning	SPARQL
Q1	Find the proteins that are in the context of gene expression.	SELECT DISTINCT ?s1 WHERE { ?t1 a genia:Protein; tao:denoted_by ?s1 . ?e1 a genia:Gene_expression . ?t1 genia:themeOf ?e1. }

Q2	Find the proteins that are in the context of localization.	SELECT DISTINCT ?s1 WHERE { ?t1 a genia:Protein; tao:denoted_by ?s1 . ?e1 a genia:Localization . ?t1 genia:themeOf ?e1. }

Q3	Find the protein that are in the context of binding.	SELECT DISTINCT ?s1 WHERE { ?t1 a genia:Protein; tao:denoted_by ?s1 . ?e1 a genia:Binding . ?t1 genia:themeOf ?e1. }

Q4	Find the protein pairs that bind to each other.	SELECT DISTINCT ?s1 ?s2 WHERE { ?t1 a genia:Protein; tao:denoted_by ?s1 . ?t2 a genia:Protein; tao:denoted_by ?s2 . ?e1 a genia:Binding . ?t1 genia:themeOf ?e1 . ?t2 genia:themeOf ?e1 . FILTER (?s1 < ?s2) }

Q5	Find the protein pairs of which one regulates the other.	SELECT DISTINCT ?s1 ?s2 WHERE { ?t1 a genia:Protein; tao:denoted_by ?s1 . ?t2 a genia:Protein; tao:denoted_by ?s2 . ?t1 genia:themeOf ?e1. ?t2 genia:causeOf ?e1. }

Q6	Find the protein pairs of which one regulates the other (transitive).	SELECT DISTINCT ?s1 ?s2 WHERE { ?t1 a genia:Protein; tao:denoted_by ?s1 . ?t2 a genia:Protein; tao:denoted_by ?s2 . ?t1 genia:themeOf+ ?e1. ?t2 genia:causeOf ?e1. }

Q7	Find the protein pairs of which one regulates expression of the other.	SELECT DISTINCT ?s1 ?s2 WHERE { ?t1 a genia:Protein; tao:denoted_by ?s1 . ?t2 a genia:Protein; tao:denoted_by ?s2 . ?e1 a genia:Gene_expression . ?t1 genia:themeOf ?e1. ?e1 genia:themeOf ?e2. ?t2 genia:causeOf ?e2. }

Q8	Find the protein pairs of which one regulates expression of the other (transitive).	SELECT DISTINCT ?s1 ?s2 WHERE { ?t1 a genia:Protein; tao:denoted_by ?s1 . ?t2 a genia:Protein; tao:denoted_by ?s2 . ?e1 a genia:Gene_expression . ?t1 genia:themeOf ?e1. ?e1 genia:themeOf+ ?e2. ?t2 genia:causeOf ?e2. }

Note that a characteristic feature of BioNLP-ST annotation is that the annotation instances are all anchored to the text pieces that refer to them, and such annotation can be considered as a semantic index to specific context. As it is a strong feature of BioNLP-ST annotation, we assume that users of the KBs induced from such annotation would want to retrieve information together with the specific parts of literature that talk about it for evidence or for the full context of the information. The SPARQL queries in the table are constructed considering that.

The first three queries, Q1, Q2, and Q3, represent simple query needs: to find proteins in the events of a specific type, *Gene_expression, Localization*, and *Binding*, respectively. Note that in the queries, the proteins and the events are to be bound to the variables **?t1 **and **?e1**, respectively, and that for the reason discussed above, they are formulated to return the text spans, to be bound to the variable **?s1**, that "denotes" the proteins.

The two queries Q3 and Q4 represent different levels of specificity of similar search needs: while Q3 is to search for single proteins in the context of binding, Q4 is to search for protein pairs binding to each other. Note that in the SPARQL construct of Q4, the two proteins bound to the variables **?t1 **and **?t2 **are themes of the event bound to **?e1**, and they need to be different to each other (stated by the FILTER constraint).

The next two queries, Q5 and Q6, are prepared to compare search performance with and without inference: both are to search for protein pairs of which one regulates the other. However, while Q6 uses transitivity reasoning (indicated by the plus sign, '+') on the predicate, genia:themeOf , Q5 does not. By using the transitivity inference, when it is known that A is a theme of B and B is a theme of C, it is assumed that A is also a theme of C.

Figure 1 shows an annotation example which can be found by Q6 but cannot be found by Q5. The protein *NFAT5 *is a theme of the Gene_expression  event represented by *expression*, and it is not retrieved by Q5. However, the Gene_expression  event is a theme of the regulation event represented by *signaling*, which is again a theme of the regulation event represented by *dependent*. By the transitivity inference, *NFAT5 *is assumed to be a theme of the regulation event represented by *dependent*. Through the process, the protein *MyD88 *can be found as a regulatory factor of *NFAT5*. As discussed in the section *Background*, alternative annotation is possible. For example, Figure 2 shows a possible variation of the annotation: it does not capture the word *dependent *as a trigger for a regulation event, but instead it connects the protein *MyD88 *as a cause to the regulation event expressed by *signaling*. Note that such an annotation variation would not be important to the domain scientists who are potential users of the KBs, but it is rather a matter of annotation guidelines and consistency. Therefore, from a KB perspective, it would be a natural demand to abstract out such an annotation difference. The transitive inference in the SPARQL query Q6 implements such a demand.

The last two queries Q7 and Q8 represent more specific query needs to search for the causal factors of proteins in a specific type of event (Gene_expression  in the queries), with and without transitivity inference.

## KB evaluation

Using the method described in the section *KB construction*, each final submission to the GE task is converted into RDF statements which are then stored in a graph. From the 10 submissions, 10 graphs are generated, each of which representing a KB to be constructed from the annotation in the corresponding submission. To represent a gold KB, a gold graph is generated from the gold annotation of the test data set, using the same method. The 11 graphs are then stored in a RDF store (specifically, Virtuoso Open Source Edition version 7.1.0 is used).

The SPARQL queries explained in the section *Queries *are submitted to the RDF store, and the results from each graph are compared to the results from the gold graph. The results are then evaluated in terms of *recall, precision *and *F1-score*.

## Comparison of the GE and GRO tasks

Table 2 shows the basic statistics of the GE and GRO datasets. The dataset of the GE task consists of full papers collected from PubMed Central [20], using the MeSH terms [21]* NF-kappa B *and *transcription factors*. The dataset of the GRO task consists of abstracts collected from PubMed [22], using a list of human transcription factors. It is thus expected that the subject domain of the two datasets would be close to each other, i.e. *NF-kappa B transcription factors *vs. *human transcription factors*, while the nature of the texts might be quite different, i.e. full papers vs. abstracts [23].

**Table 2:** Basic statistics of GE'13 and GRO'13 benchmark datasets.

	GE'13				GRO'13	
	**Train**.	**Devel**.	**Test**	**Train**.	**Devel**.	**Test**

Documents	10 papers	10 papers	14 papers	150 abstracts	50 abstracts	100 abstracts
Entities	3692	4452	4686	5902	1910	4007
Events	2817	3199	3348	2175	747	2319

We tested whether the dataset of the GE task can be used to improve the performance of participant systems when it is converted into the GRO task format, and vice versa. We converted the datasets of the two tasks into each other's format to measure the overlap of the two tasks in terms of corpus annotations as well. This may help lead us to a unified framework for the shared tasks. We performed the conversion via equivalence mappings between the concepts of the two task ontologies. Table 3 shows the equivalence mappings used for the conversion. In fact, the GE'13 corpora also used the concepts of Deacetylation and Ubiquitination, but we ignored them since there is no correspondent in GRO for them. Note that we mapped Binding from Genia to BindingToProtein from GRO, because all participants of Binding events in the GE'13 corpora are proteins.

**Table 3:** Mappings between the Genia ontology concepts for the GE'13 task and the GRO concepts for the GRO'13 task.

Genia concept	GRO concept
Acetylation	Acetylation
Binding	BindingToProtein
Gene_expression	GeneExpression
Localization	Localization
Negative_regulation	NegativeRegulation
Phosphorylation	Phosphorylation
Positive_regulation	PositiveRegulation
Protein	Gene
Protein	Protein
Protein_catabolism	ProteinCatabolism
Protein_modification	ProteinModification
Regulation	RegulatoryProcess
Transcription	Transcription

We converted the training data of the two tasks into each other's format according to the mappings in Table 3. When converting the GE data to the GRO task, the Genia concepts are replaced with the corresponding GRO concepts according to the table. When converting the GRO data to the GE task, not only the GRO concepts found in the table, but also those whose ancestors have mappings to the Genia concepts, are converted. For example, an instance of the GRO concept *RegulationOfGeneExpression *is converted to an instance of the Genia concept *Regulation*, since *RegulationOf GeneExpression *is a subconcept of the GRO concept *RegulatoryProcess*, which is equivalent to *Regulation*.

We used the converted data for increasing the training data set size of the two tasks, especially where the GRO task's participants suffered from the relatively low amount of training data. For example, we combined the GRO task training data and the conversion of the GE task training data and used them for training the TEES system [24] on the GRO task. We measured the system performance before and after the data conversion to see its effect and analyzed the results. We also tested the other conversion, and with another event extraction system as reported in the next section.

## Results and discussions

### Results of KB-oriented evaluation

This section reports the results of the KB-oriented evaluation that has been carried out on the KBs induced from the final submissions to the GE task in 2013. For the purpose of comparison, the results of annotation-oriented evaluation on the four event types, *Gene-expression, Localization, Binding*, and *REGULATION-ALL *(which subsumes all the regulation types, *Regulation, Positive- *and *Negative-regulation)*, are shown in Tables 4 and 5.

**Table 4:** Results of annotation-oriented evaluation on *Gene-expression *and *Localization*. Acronyms: GS=gold standard, P=positives, TP=true positives, R=recall, P=precision, F=f-score.

	Gene_expression			Localization		
	**P**	**TP**	**R / P / F**	**P**	**TP**	**R / P / F**

*GS*	619	619		99	99	-
EVEX	600	504	81.42 / 84.00 / 82.69	56	47	47.47 / 83.93 / 60.65
TEES-2.1	600	504	81.42 / 84.00 / 82.69	59	50	**50.51 / 84.75 / 63.29**
BioSEM	526	457	73.83 / 86.88 / 79.83	47	42	42.42 / 89.36 / 57.53
NCBI	641	495	79.97 / 77.22 / 78.57	47	39	39.39 / 82.98 / 53.42
DlutNLP	580	480	77.54 / 82.76 / 80.07	39	35	35.35 / 89.74 / 50.72
HDS4NLP	556	501	**80.94 / 90.11 / 85.28**	66	50	50.51 / 75.76 / 60.61
NICTANLM	761	506	81.74 / 66.49 / 73.33	52	31	31.31 / 59.62 / 41.06
USheff	450	386	62.36 / 85.78 / 72.22	27	23	23.23 / 85.19 / 36.51
UZH	497	406	65.59 / 81.69 / 72.76	39	34	34.34 / 87.18 / 49.28
HCMUS	790	488	78.84 / 61.77 / 69.27	61	32	32.32 / 52.46 / 40.00

**Table 5:** Results of annotation-oriented evaluation on *Binding *and *REGULATION-ALL*. Acronyms: GS=gold standard, P=positives, TP=true positives, R=recall, P=precision, F=f-score.

	Binding			REGULATION_ALL		
	**P**	**TP**	**R / P / F**	**P**	**TP**	**R / P / F**

*GS*	333	333	-	1944	1944	-
EVEX	306	137	41.14 / 44.77 / 42.88	1336	630	**32.41 / 47.16 / 38.41**
TEES-2.1	318	141	**42.34 / 44.34 / 43.32**	1436	643	33.08 / 44.78 / 38.05
BioSEM	302	158	47.45 / 52.32 / 49.76	1115	547	28.19 / 49.06 / 35.80
NCBI	299	125	37.54 / 41.81 / 39.56	865	481	24.74 / 55.61 / 34.25
DlutNLP	308	136	40.84 / 44.16 / 42.43	1185	515	26.49 / 43.46 / 32.92
HDS4NLP	412	139	41.74 / 33.74 / 37.32	780	411	21.14 / 52.69 / 30.18
NICTANLM	344	107	32.13 / 31.10 / 31.61	891	420	21.60 / 47.14 / 29.63
USheff	224	105	31.53 / 46.88 / 37.70	1050	324	16.67 / 30.86 / 21.64
UZH	264	74	22.22 / 28.03 / 24.79	1912	381	19.60 / 19.93 / 19.76
HCMUS	478	129	38.74 / 26.99 / 31.81	693	215	11.06 / 31.02 / 16.31

Table 6 shows the results for the queries Q1 and Q2. Each row in the table shows the result from the KB induced from the annotation submitted by the team indicated by the label in the first column. Note that *GS *means the KB induced from the gold annotation. With these simple queries, which do not require abstraction or inference, the results are similar to the results from the annotation-oriented evaluation. There is a small difference in the number of true positives, which is because the annotation-oriented evaluation counts the number of "events", while the KB-oriented evaluation counts the number of proteins involved in the events.

**Table 6:** Results of KB-oriented evaluation for Q1 (Find the proteins in the context of gene expression) and Q2 (Find the proteins in the context of localization).

	Q1 (Gene_expression)			Q2 (Localization)		
	**P**	**TP**	**R / P / F**	**P**	**TP**	**R / P / F**

*GS*	604	604	-	94	94	-
EVEX	604	497	82.28 / 82.28 / 82.28	56	45	47.87 / 80.36 / 60.00
TEES-2.1	604	497	82.28 / 82.28 / 82.28	59	48	**51.06 / 81.36 / 62.75**
BioSEM	537	456	75.50 / 84.92 / 79.93	52	43	45.74 / 82.69 / 58.90
NCBI	647	493	81.62 / 76.20 / 78.82	46	38	40.43 / 82.61 / 54.29
DlutNLP	591	475	78.64 / 80.37 / 79.50	38	35	37.23 / 92.11 / 53.03
HDS4NLP	563	500	**82.78 / 88.81 / 85.69**	68	50	53.19 / 73.53 / 61.73
NICTANLM	748	501	82.95 / 66.98 / 74.11	52	30	31.91 / 57.69 / 41.10
USheff	452	386	63.91 / 85.40 / 73.11	26	23	24.47 / 88.46 / 38.33
UZH	496	404	66.89 / 81.45 / 73.45	40	34	36.17 / 85.00 / 50.75
HCMUS	763	481	79.64 / 63.04 / 70.37	68	31	32.98 / 45.59 / 38.27

Acronyms: GS=gold standard, P=positives, TP=true positives, R=recall, P=precision, F=f-score.

Table 7 shows how much the performance drops when pairs of binding proteins are required to be retrieved instead of single proteins. The performance drop is substantial, even considering that the complexity is expected to be quadratic: when the performance of finding single proteins is *P*, the performance of finding pairs is expected to be *P × P*, e.g., 59.12% × 59.12% = 34.95% vs. 28.19% in the case of *TEES-2.1*, and 63.20% × 63.20% = 39.94% vs. 19.35% in the case of *HDS4NLP*. This is because even after two proteins are correctly found to be connected to the same trigger indicating the event *Binding*, it still needs to be determined whether the two proteins are involved in a single binding event *(collective parsing) *or in two separate events *(distributive parsing)*. Note that the terms, *collective *and *distributive parsing*, are inspired from the linguistic terms *collective *and *distributive reading*. The results indicate that the system *HDS4NLP *is generally good at extracting individual predicate-argument relations (see also Table 6), while *TEES-2.1 *does a much better job in determining collective vs. distributive parsing.

**Table 7:** Results of KB-oriented evaluation for Q3 (Find the protein in the context of binding) and Q4 (Find the protein pairs binding to each other).

	Q4 (pair Binding)			Q4 (pair Binding)		
	**P**	**TP**	**R / P / F**	**P**	**TP**	**R / P / F**

*GS*	300	300	-	83	83	-
EVEX	324	182	60.67 / 56.17 / 58.33	122	27	32.53 / 22.13 / 26.34
TEES-2.1	336	188	62.67 / 55.95 / 59.12	144	32	**38.55 / 22.22 / 28.19**
BioSEM	355	182	60.67 / 51.27 / 55.57	114	21	25.30 / 18.42 / 21.32
NCBI	318	177	59.00 / 55.66 / 57.28	167	24	28.92 / 14.37 / 19.20
DlutNLP	352	193	64.33 / 54.83 / 59.20	179	25	30.12 / 13.97 / 19.08
HDS4NLP	393	219	**73.00 / 55.73 / 63.20**	72	15	18.07 / 20.83 / 19.35
NICTANLM	369	175	58.33 / 47.43 / 52.32	177	21	25.30 / 11.86 / 16.15
USheff	252	156	52.00 / 61.90 / 56.52	43	13	15.66 / 30.23 / 20.63
UZH	255	143	47.67 / 56.08 / 51.53	0	0	00.00 / 00.00 / 00.00
HCMUS	491	207	69.00 / 42.16 / 52.34	75	19	22.89 / 25.33 / 24.05

Acronyms: GS=gold standard, P=positives, TP=true positives, R=recall, P=precision, F=f-score.

Table 8 shows how much the performance changes when transitive inference is used to abstract out the schematic difference in annotations, which is a concern of annotation-oriented evaluation, but not of KB-oriented evaluation. Some systems exhibit much better performance in KB-oriented evaluation than in annotation- oriented evaluation, e.g., *TEES-2.1 *(20.81% → 46.02%), *NCBI *(11.27% → 29.81%), and *NICTANLM *(7.19% → 27.12%). Note that regulation events in the GE task annotation are represented in a recursive manner, which may require more computation than that for simple type events. When the top two systems, *EVEX *and *TEES-2.1*, are compared, it may be said that *EVEX *is more optimized to the annotation-oriented evaluation, which is the original official evaluation of the GE task. Note that it is often the case for a retraining approach to be optimized to the object function. It is notable that while in the context of automatic annotation, the performance with regulation-type events looks almost too poor to be useful, in the context of KB application, it is much more encouraging, though not sufficient. The observation in Table 9 is similar to that in Table 8 as the contrast may be less dramatic.

**Table 8:** Results of KB evaluation for Q5 (Find the protein pairs of which one regulates the other) and Q6 (Find the protein pairs of which one regulates the other, transitive).

	Q5 (Regulation)			Q6 (transitive Regulation)		
	**P**	**TP**	**R / P / F**	**P**	**TP**	**R / P / F**

*GS*	108	108	-	360	360	-
EVEX	61	18	16.67 / 29.51 / 21.30	197	126	35.00 / 63.96 / 45.24
TEES-2.1	65	18	16.67 / 27.69 / 20.81	218	133	**36.94 / 61.01 / 46.02**
BioSEM	45	14	12.96 / 31.11 / 18.30	155	90	25.00 / 58.06 / 34.95
NCBI	34	8	07.41 / 23.53 / 11.27	103	69	19.17 / 66.99 / 29.81
DlutNLP	69	20	**18.52 / 28.99 / 22.60**	174	106	29.44 / 60.92 / 39.70
HDS4NLP	31	13	12.04 / 41.94 / 18.71	31	17	04.72 / 54.84 / 08.70
NICTANLM	31	5	04.63 / 16.13 / 07.19	112	64	17.78 / 57.14 / 27.12
USheff	18	5	04.63 / 27.78 / 07.94	60	35	09.72 / 58.33 / 16.67
UZH	6	0	00.00 / 00.00 / 00.00	21	8	02.22 / 38.10 / 04.20
HCMUS	94	9	08.33 / 09.57 / 08.91	156	33	09.17 / 21.15 / 12.79

Acronyms: GS=gold standard, P=positives, TP=true positives, R=recall, P=precision, F=f-score.

**Table 9:** Results of KB evaluation for Q7 (Find the protein pairs of which one regulates expression of the other) and Q8 (Find the protein pairs of which one regulates expression of the other, transitive).

	Q7 (Regulation of Exp)			Q8 (transitive Regulation of Exp)		
	**P**	**TP**	**R / P / F**	**P**	**TP**	**R / P / F**

*GS*	111	111	-	128	128	-
EVEX	52	38	34.23 / 73.08 / 46.63	61	50	39.06 / 81.97 / 52.91
TEES-2.1	67	43	**38.74 / 64.18 / 48.31**	77	56	**43.75 / 72.73 / 54.63**
BioSEM	26	21	18.92 / 80.77 / 30.66	31	25	19.53 / 80.65 / 31.45
NCBI	37	25	22.52 / 67.57 / 33.78	37	32	25.00 / 86.49 / 38.79
DlutNLP	49	36	32.43 / 73.47 / 45.00	52	41	32.03 / 78.85 / 45.56
HDS4NLP	0	0	00.00 / 00.00 / 00.00	0	0	00.00 / 00.00 / 00.00
NICTANLM	42	24	21.62 / 57.14 / 31.37	40	30	23.44 / 75.00 / 35.71
USheff	31	20	18.02 / 64.52 / 28.17	29	20	15.63 / 68.97 / 25.48
UZH	10	2	01.80 / 20.00 / 03.31	10	3	02.34 / 30.00 / 04.35
HCMUS	38	16	14.41 / 42.11 / 21.48	38	16	12.50 / 42.11 / 19.28

Acronyms: GS=gold standard, P=positives, TP=true positives, R=recall, P=precision, F=f-score, Exp=Gene_expression.

The experimental results of the extended evaluation provide additional insight to the performance of the systems. For example, while *EVEX *is evaluated to perform best for production of gold annotation. *TEES-2.1 *is evaluated better when the application is KB construction.

## Results of task dataset conversion

Table 10 shows the statistics of the conversion using the equivalence correspondences. As shown in the table, most of the entities and events of the GE data are convertible to the GRO task, while many of the entity events of the GRO data are not. Table 11 shows the most frequent GRO concepts that correspond to Genia concepts and their ancestor concepts. Note that among the 4,193 convertible entities, 1,042 entities (24.9%) are converted via proper subsump- tion (or non-equivalence) relations between the two ontologies, e.g., *Protein > TranscriptionFactor*, where *A > B *indicates the concept *A *subsumes the concept *B*; in other words, *B *is a subconcept of A. Table 12 shows the most frequent GRO concepts that are not convertible to the GE task, including those that indicate where the gene regulation events take place (e.g. organism, tissue, cell), DNA without specific location of interest, protein domains, chemicals, quantitative changes without clear causal effects, and disease.

**Table 10:** Statistics of conversion rates.

		GE → GRO	GRO → GE
Entities	Convertible	6,449 (98.1%)	4,193 (51.9%)
	Non-convertible	125 (1.9%)	3,881 (48.1%)

Events	Convertible	3,436 (92.5%)	1,094 (25.5%)
	Non-convertible	280 (7.5%)	3,188 (74.6%)

**Table 11:** Most frequent GRO concepts and their ancestor concepts that correspond to Genia concepts.

Genia concept (count)	GRO concepts and their ancestors corresponding to the Genia concept (count)
Protein (2887)	Protein (1521)
	Gene (482)
	TranscriptionFactor < TranscriptionRegulator < Protein (294)
	Enzyme < Protein (264)
	ProteinSubunit < Protein (143)

Regulation (289)	RegulatoryProcess (221)
	PositiveRegulationOfGeneExpression < RegulationOfGeneExpression < RegulatoryProcess (22)
	NegativeRegulationOfTranscription < RegulationOfTranscription < RegulatoryProcess (18)

Gene_expression (237)	GeneExpression (237)

Positive_regulation (229)	PositiveRegulation (229)

Negative_regulation (145)	NegativeRegulation (145)

Binding (126)	BindingToProtein (126)

Localization (112)	Localization (62)
	Transport < Localization (36)
	ProteinTargeting < ProteinTransport < Localization (12)

Transcription (105)	Transcription (83)
	TranscriptionOfGene < Transcription (22)

**Table 12:** Most frequent GRO concepts that are not convertible to Genia.

Level 3	Level 4	Level 5	Level 6	Count
(under the branch of Continuant > PhysicalContinuant)				
LivingEntity	> Organism	> Eukaryote		470
LivingEntity	> Cell			383
Tissue				218
MolecularEntity	> InformationBiopolymer	> NucleicAcid	> DNA	193
MolecularEntity	> InformationBiopolymer	> ProteinDomain		171
MolecularEntity	> Chemical	> OrganicChemical	> AminoAcid	129
CellComponent				122

(under the branch of Occurrent > Process)				
Increase				92
Disease				91
PhysicalInteraction	> Binding	> BindingOfProteinToDNA		71
MolecularProcess	> Pathway	> SignalingPathway		67
Mutation				47

The count of the last column is the count of the concept at the lowest level in each row.

We converted the training data of each task to the other task format and used the converted data as additional training data for the latter task. For example, we converted the GRO task training data to the GE task format, used it together with the original training data of the GE task to train the TEES system on the GE task, and evaluated the system against the GE task test data. We followed the same procedure for the GE->GRO conversion. We used the default settings of version 2.1.1 of the system [25].

The GRO→GE conversion (i.e. using the converted GRO data as additional training data for the GE task) resulted in an increase of the performance from 38.2 F-score to 42.2 F-score. The conversion enhanced the system performance in most of the event classes, which may mean that the GE task requires more training data to saturate the system performance and that the class (or concept) distribution of the convertible data of the GRO task is not heavily biased in comparison with the GE task.

However, the GE→GRO shows a slightly negative effect on the performance of the TEES system: The original performance of the system in terms of F-measure was 24.9%, while it shows 24.0% F-measure with the additional data from the GE task, dropping F-measure by 0.9 percentage points. Table 13 shows the performance change for some individual GRO concepts by the GE->GRO conversion. The first five concepts in the table are those whose instances are increased by the data conversion, but whose performance changes are below 5 percentage points. The last four concepts in the table are those whose performance has changed by more than 5 percentage points and from which true positives identified by the system before the data conversion were above 10. Note that all these four concepts do not obtain any new instances from the data conversion. As shown in the table, all the four concepts show a performance drop.

**Table 13:** Performance changes for different GRO concepts after using the additional converted data from the GE task.

Concept	No. of instances of concept in the original training dataset of GRO'13	No. of instances of concept converted from the GE'13 training dataset	F-measure before conversion integration	F-measure after conversion integration	Change
GeneExpression	221	748	58.8%	63.7%	4.9%
PositiveRegulation	206	785	16.3%	13.7%	−2.6%
RegulatoryProcess	183	305	24.1%	23.7%	−0.4%
NegativeRegulation	124	512	16.5%	16.1%	−0.4%
BindingToProtein	126	201	32.7%	32.1%	−0.6%

SignalingPathway	66	0	54.6%	35.3%	−19.3%
CellGrowth	17	0	32.3%	26.4%	−5.9%
Mutation	45	0	23.3%	17.9%	−5.4%
Disease	91	0	19.0%	10.1%	−8.9%

This performance difference between the concepts that obtained more instances from the data conversion and those that did not may be due to the following factors: 1) The five GRO concepts populated with additional instances from the GE task are already highly populated in the original GRO corpora, and thus their performance is not affected much. The average number of instances for the populated event concepts in the GRO'13 training data is 48, while that of all event concepts is only 13 (see Table 14). 2) The data conversion increases the imbalance of instances among GRO concepts and causes overfitting and thereby performance drop. The average number of instances per event concept that are converted from the GE'13 training data is 287, which is larger than the overall average 13 (see Table 14). 3) The different styles of annotation between the two tasks [26] may lead to heterogeneity of the combined data and thereby to little synergistic gain.

**Table 14:** Average number of instances per ontology concept.

(per applicable concept)	GE	GRO
Average number of instances	82	13
Average number of convertible instances to the other task	287	47

We also examined whether the differences between the two ontologies of the two tasks show specific impact on system performance. We considered two differences: 1) GRO differentiates between Gene and Protein, while the Genia ontology has only the Protein concept; and 2) GRO has more specific subconcepts of events than Genia (e.g. PositiveRegulationOfGeneExpression < PositiveRegulation). First, as shown in Table 11, 1,521 instances of the GRO concept "Protein" and 482 instances of the GRO concept "Gene" were converted to the Genia concept "Protein". If we only consider the GRO events at least one of whose participants is a Gene instance or a Protein instance, 570 events with a Protein instance and 116 events with a Gene instance were converted to the corresponding Genia events with Protein instances. We assume that these ratios of 25% (entity) and 17% (event) of Gene- specific information in the GRO dataset would be similarly found in the GE dataset, since the two datasets have large overlaps. Second, as shown in Table 11, around 90% of the GRO event instances are mapped to the equivalent Genia concepts, while the rest belong to the GRO concepts that are more specific than the mapped Genia concepts. In short, the conversion of the GE data to the GRO format ignores the fact that 10%-17% of the event instances should be mapped to more specific concepts.

We compared the changes in errors (i.e. false positives, false negatives) of events that are related to the differences by the data conversion. Table 15 shows the number of errors in the events at least one of whose participants (i.e. agent, patient) is either a Gene or Protein instance before and after the data conversion. As shown in the table, it is not clear if the data conversion has a positive or negative effect on the system performance. Table 16 shows the number of errors in the events of frequent subconcepts of the GRO concepts that have equivalent Genia concepts (e.g. PositiveRegulation). This table also does not show any definite effect of the data conversion. These indefinite results may be due to the small ratios of the affected instances.

**Table 15:** Changes of errors in events with at least one Gene/Protein participant by the data conversion

	Before conversion		After conversion	
	FP	FN	FP	FN

Protein	85	121	67	125
Gene	26	58	31	57

**Table 16:** Changes of errors for frequent subconcepts of the GRO concepts that have equivalent correspondent Genia concepts, by the data conversion.

Concept	No. of instances	Before conversion		After conversion	
		**FP**	**FN**	**FP**	**FN**

BindingOfProteinToDNA	55	42	45	34	46
PositiveRegulationOfGeneExpression	33	11	25	6	28
Heterodimerization	32	6	25	4	25
BindingOfTranscriptionFactorToDNA	25	0	25	0	25
PositiveRegulationOfTranscription	24	0	4	0	4

FP stands for false positives and FN stands for false negatives.

We also tested the effect of the GE→GRO conversion on a rule-based system [27]. (TEES is based on machine learning.) The rule-based system was developed for populating the *E. coli *transcription regulatory database RegulonDB, and its results can be used for the GRO task evaluation since the system represents all of the events for database population with the GRO. Note, however, that this experiment is preliminary since the system has not been seriously adapted for the GRO task. The rule-based system also shows a slight performance drop (from 16.3% to 15.2%). The highest performance gain was made for the concept ProteinCatabolism (from 0% to 33.3%; 7 true instances), which was benefited by the data conversion (see Table 3), while the largest performance drop was seen for BindingOfTranscriptionFactor (from 19.4% to 6.5%; 25 true instances), which was not benefited by the data conversion.

## Conclusions

The third edition of the BioNLP-ST was organized with the grand theme of *knowledge base construction*, in order to extend the potential applications of the tasks by more carefully considering the perspective of domain scientists. The paper presents an extended evaluation - the KB-oriented evaluation - of the GE task, to better fulfill the grand theme. Experimental results suggest that the participating systems may be evaluated differently in different application contexts, annotation vs. KB.

The paper also presents a comparison of the GE task to the GRO task toward a KB with richer semantics. The inter-task resource conversion was found useful only when the converted data did not bias the class distribution of the original data. In the case of GE→GRO conversion, it could not improve either a machine learning-based system or a rule-based system.

As future work, the KB-oriented evaluation will be made publicly available as an automatic online service, so that the participants of the task can consider the aspect of KB-oriented evaluation during their system development. While the evaluation was carried out as an intrinsic evaluation, exploring its connection to a relevant extrinsic evaluation, e.g., Task 2 (Biomedical question answering over interlinked data) of Question Answering over Linked Data (QALD)-4 [28], should be beneficial. Also, the comparison, and eventually an integration, of the GE and GRO tasks will be explored toward information extraction (IE) for KB with richer semantics.

## Competing interests

The authors declare that they have no competing interests.

## Authors' contributions

JDK designed and implemented the KB-evaluation with the GE task. JJK and DRS conceived the comparison of the GRO and GE tasks. JJK and XH designed and implemented the comparison and analysis. All the authors read and approved the final version of the manuscript.

## Declarations

This work has been funded partly by JST (Japan Science and Technology Agency) under the Life Science Database Integration Project, and partly by the EU STREP grant 028099 ("BOOTStrep") under the 6th EU Framework Programme. Publication costs for this article are funded by JST

This article has been published as part of *BMC Bioinformatics *Volume 16 Supplement 10, 2015: BioNLP Shared Task 2013: Part 1. The full contents of the supplement are available online at http://www.biomedcentral.com/bmcbioinformatics/supplements/16/S10.
